# Transcriptomic profiling of Pseudomonas migulae revealed gene regulatory properties during biodegradation of aromatic hydrocarbons under cold stress

**DOI:** 10.1099/mgen.0.001470

**Published:** 2025-09-16

**Authors:** Hiroki Yanagita, Robert A. Kanaly, Jiro F. Mori

**Affiliations:** 1 Graduate School of Nanobioscience, Yokohama City University, Kanagawa, Japan

**Keywords:** aromatic hydrocarbon biodegradation, cold adaptation, *Pseudomonas*, transcriptomics

## Abstract

Bacterial strains within the genus *Pseudomonas* have often been characterized for their ability to biodegrade aromatic hydrocarbon pollutants, as well as high adaptability to diverse environmental stresses, including cold stress. In this study, a newly isolated bacterium, *Pseudomonas migulae* strain HY-2, which can grow at 10 °C by utilizing the aromatic hydrocarbon pollutant *p*-hydroxybenzoic acid (PHBA) as the sole carbon source, was employed as a model to investigate bacterial gene regulatory properties during cold stress adaptation and aromatic hydrocarbon biodegradation. Complete genome sequencing and transcriptomic analysis under cold conditions revealed that strain HY-2 up-regulates at least ten types of chaperone proteins potentially involved in transcriptional, translational and post-translational regulation to maintain protein homeostasis. Transcriptomic data also suggested that cold-induced biofilm formation was enhanced by exogenous polyamines, spermidine or putrescine, which was confirmed through subsequent bioassays. It is hypothesized that exogenous polyamines, commonly supplied by surrounding organisms, enhance biofilm formation in *Pseudomonas*, thereby aiding in cold stress resistance. Notably, expressions of functional genes responsible for PHBA biodegradation in strain HY-2 were mostly unaffected by cold stress – only three genes, including a PHBA transporter gene, were significantly up-regulated while the neighbouring 10 genes were unaffected – resulting in minimal impact on growth efficiency at 10 °C. These findings provide new insights into the genetic properties of cold adaptation and stress resilience in *Pseudomonas* and reaffirm the potential of this genus for bioremediation applications in natural environments without the need for extensive anthropogenic intervention.

**Impact Statement**

The application of micro-organisms for pollutant bioremediation in natural environments often encounters multiple stress factors, including non-optimal low temperatures. Therefore, biotechnologically useful micro-organisms are expected to possess the ability to withstand such environmental stressors. This study presents a transcriptomic investigation of a newly isolated *Pseudomonas* strain capable of biodegrading aromatic hydrocarbon pollutants under cold stress (<10 °C), revealing detailed gene regulatory mechanisms that support adaptation to low temperatures during pollutant biodegradation. The insights gained from this study shall be valuable for the future biotechnological application of *Pseudomonas* strains.

## Data Summary

The genome sequence of strain HY-2 is available in the National Center for Biotechnology Information (NCBI) GenBank under the accession number CP171224 and the IMG/MER database under accession number 8103273928. The raw genome sequence data and raw RNA-seq data are available in the NCBI Sequence Read Archive under accession numbers PRJNA1170499 and PRJNA1240940.

## Introduction

Multiple physicochemical environmental stress factors – such as non-optimal temperature, pH and salinity – influence the stability and efficiency of microbial biodegradation of environmental pollutants [1,2]. Therefore, upon the application of micro-organisms for pollutant bioremediation at field sites, they are expected to possess the ability to cope not only with the toxicity of pollutants but also with these environmental stressors. Although well-studied model micro-organisms are mostly characterized as mesophiles that optimally grow at temperatures between 20 and 45 °C, the temperature of surface freshwater bodies on Earth is typically below 15 °C and fluctuates seasonally [3]. Under cold temperature stress, micro-organisms face decreased membrane fluidity, reduced efficiency of mRNA transcription and translation due to increased stability of RNA and DNA secondary structures and impaired functions of proteins and ribosomes [4]. Furthermore, cold stress additionally induces intracellular oxidative stress due to elevated levels of reactive oxygen species, which result from increased oxygen solubility at low temperatures [5,6].

Bacterial strains within the genus *Pseudomonas*, which have been studied as model organisms for the capacity to biodegrade aromatic hydrocarbon pollutants [7,10], have been widely isolated from various environments (terrestrial and aquatic habitats) and have repeatedly been reported to withstand a variety of external stresses [11,13]. In the clinical research field, strains of *Pseudomonas aeruginosa*, an opportunistic human pathogen, have been studied for their remarkable antibiotic resistance, and the genomic features corresponding to this ability have been extensively investigated [13,14]. Regarding cold stress resistance, a model strain, *Pseudomonas putida* strain KT2440, has been reported to grow at cold temperature (10 °C), while it optimally grows at 30 °C; a previous transcriptomic and proteomic investigation analysing the initial response of this strain to the sudden drop in temperature revealed that it adapts to cold stress by activating ribosome-associated proteins that may facilitate translational efficiency, as well as the outer membrane proteins potentially involved in anabolic and catabolic pathways [15]. A recent study on *Pseudomonas fluorescens* strain S01, which is capable of biodegrading polycyclic aromatic hydrocarbons as well as growing at 5–40 °C, reported enhanced activities of methyl-accepting chemotaxis proteins and biofilm formation during growth at 15 °C compared to 25 °C [16]. However, these previous studies have not provided a comprehensive understanding of the gene regulatory properties that enable *Pseudomonas* to maintain growth and aromatic hydrocarbon biodegradation capabilities while adapting to cold stress conditions, which may be valuable for future biotechnological applications of this bacterial genus.

In this study, a newly isolated *Pseudomonas* bacterium, *Pseudomonas migulae* strain HY-2, capable of growing at cold temperatures (< 10 °C) while utilizing the common aromatic hydrocarbon environmental pollutant *p*-hydroxybenzoic acid (PHBA) as the sole carbon source, was employed as a new model micro-organism of *Pseudomonas* to investigate cold adaptation mechanisms during the biodegradation of aromatic hydrocarbon pollutants. To achieve this, complete genome sequencing and detailed transcriptomic analysis of strain HY-2 under cold stress in the presence of PHBA were conducted. The results obtained here identified multiple proteins potentially responsible for transcriptional, translational and post-translational regulation to maintain metabolic homeostasis, as well as a polyamine-induced biofilm formation system in response to cold stress conditions.

## Methods

### Chemicals

*p*-Hydroxybenzoic acid (>99% purity), *p*-coumaric acid (>98% purity) and phenanthrene (98% purity) were purchased from Sigma-Aldrich (St. Louis, MO, USA). *N*,*N*-Dimethylformamide (DMF) (>99% purity), ethyl acetate (HPLC grade), methanol (liquid chromatography MS grade), spermidine (>95% purity) and putrescine (>98% purity) were purchased from Wako Chemical (Osaka, Japan).

### Bacterial isolation and culture conditions of P. migulae strain HY-2

*P. migulae* strain HY-2 was isolated from a bacterial enrichment culture grown on phenanthrene, a three-ring polycyclic aromatic hydrocarbon. These bacteria originated from non-polluted groundwater outflow sediment collected in Sakae-ku, Yokohama, Japan, in May 2021, as part of a study on local micro-organisms involved in metal cycling in the outflow [17,18]. At the time of collection, the water temperature was 11.2 °C, and the sediment was collected in a centrifugation tube and stored at 4 °C for over 2 years before it was subjected to the subsequent experiments. Bacteria from the sediment were resuspended in sterilized water, inoculated into Stanier’s basal medium (SBM) [19], which was used in past studies on aromatic hydrocarbon-degrading bacteria [9,20,22]. Cells were cultured with 50 mg l^−1^ phenanthrene as the sole carbon source by rotary shaking at 150 r.p.m. in the dark at 30 °C and were sub-cultured once per month. From this bacterial consortium, two *Pseudomonas* strains were isolated as colonies on Luria-Bertani (LB) agar. Among these, strain HY-2 was further maintained as a pure culture in SBM with 100 mg l^−1^ PHBA (in DMF prior to addition), a potential phenanthrene biodegradation product, as the sole carbon source.

### Evaluation of the PHBA biodegradation ability of strain HY-2 at cold temperature

The biodegradation ability of strain HY-2 for PHBA was assessed at both optimal (30 °C) and cold (10 °C) temperatures. Bacterial cells pre-grown for 5 days with 100 mg l^−1^ PHBA at 30 °C were transferred to fresh SBM containing either 500 mg l^−1^ PHBA or no carbon source and further incubated at either 30 °C or 10 °C with rotary shaking at 150 r.p.m. in the dark, in triplicate for each condition. Bacterial growth was monitored by measuring OD_600_ every 4–12 h using a spectrophotometer, by taking a small aliquot from each culture at each time point. Residual PHBA in the cultures was extracted using liquid-liquid extraction with equal volumes of ethyl acetate [21,22]. The organic phase containing PHBA was dried at 30 °C by flushing with nitrogen gas, and the residues were resuspended in methanol to a 1.5 ml volume in brown glass vials. Extracts were analysed by liquid chromatography (Waters 2690 Separations Module, Waters, MA, USA) using a Capcell Pak C18 column (2.0 mm i.d. × 50 mm, 3.0 µm particle size; Osaka Soda, Osaka, Japan) in-line with a Waters 2998 diode array detector. PHBA was separated with a mobile phase that consisted of 2% (v/v) acetic acid in water and methanol (20:80) at a flow rate of 0.1 ml min^−1^. PHBA was quantified by UV detection at 254 nm, with *p*-coumaric acid as the internal standard, which was added to the extracts at a concentration of 50 mg l^−1^.

### Genome sequencing of strain HY-2

Genomic DNA of strain HY-2 was extracted from bacterial cells grown overnight in LB liquid medium using the NucleoBond high-molecular-weight DNA Kit (Macherey-Nagel, Düren, Germany) and further purified using DNA Clean Beads (MGI Tech, Shenzhen, China) [23,24]. After DNA shearing (~10–20 kbp) with a g-TUBE (Covaris, Woburn, MA, USA), the SMRTbell template libraries were prepared with the SMRTbell Express Template Prep Kit 2.0 (PacBio, Menlo Park, CA, USA) and were sequenced on the Revio platform using the Revio polymerase kit (PacBio). Through adapter trimming and quality filtering of raw reads using SMRT Link (ver. 13.0.0.207600) and Filtlong (ver. 0.2.1), HiFi reads with a length of >1,000 bp were generated. *De novo* assembly was performed using Flye (ver. 2.9.2-b1786) [25], which was followed by evaluation of genome completeness using CheckM (ver. 1.2.2) [26] and chromosome circular validation using Bandage (ver. 0.8.1) [27]. Gene annotation was performed with theNational Center for Biotechnology Information (NCBI) Prokaryotic Genome Annotation Pipeline (ver. 6.8) and the IMG Annotation Pipeline (ver. 5.2.1). Average nucleic acid identity (ANI) between the strain HY-2 genome and reference genomes was determined using fastANI (ver. 1.33) [28]. All software was used with default settings unless otherwise specified.

### RNA-seq transcriptomic profiling of strain HY-2 that grew on PHBA at cold temperature

Bacterial cells of strain HY-2 grown on 500 mg l^−1^ PHBA at either 30 °C or 10 °C were harvested at the exponential phase (OD_600_=0.20) for RNA extraction, in triplicate for each condition. Total RNA was extracted using the NuleoSpin RNA Kit (Macherey-Nagel), and ribosomal RNA was removed with the Ribo-Zero Plus rRNA Depletion Kit (Illumina, San Diego, CA, USA). Strand-specific cDNA libraries were prepared using the NEBNext Ultra II Directional RNA Library Prep Kit (New England Biolabs, Beverly, MA, USA) and were sequenced on the Illumina NovaSeq X Plus platform (Illumina) (2×150 bp paired-end). After adapter trimming and quality filtering of raw reads using Fastp (ver. 0.23.4) [29], the high-quality reads were mapped to the assembled genome of strain HY-2 using Bowtie2 (ver. 2.5.1) [30] with default settings for coverage calculation. Read counts of each coding sequence were determined with featureCounts (ver. 2.0.6) [31]. Differentially expressed genes (DEGs) between cultures grown at 30 and 10 °C were identified using the R-based tool edgeR package (ver. 4.2.1) [32], according to a false discovery rate (FDR) threshold and log_2_ fold change (log_2_FC) filtering. A volcano plot of DEGs was generated using the EnhancedVolcano package (ver. 1.22.0) in R.

### Biofilm formation assay

Biofilm formation of strain HY-2 was quantified using the crystal violet biofilm assay [33]. Strain HY-2 cells pre-grown for 5 days with PHBA at 30 °C were transferred to 2 ml of fresh SBM medium in a 10-ml glass tube containing 500 mg l^−1^ PHBA and further incubated statically at either 30 °C or 10 °C in the dark. After incubation, the cultures were discarded, and the tubes were gently rinsed three times with distilled water. Biofilms adhered to the tube walls were stained with 0.1% crystal violet (Becton Dickinson, Sparks, MD, USA) and then washed three times with distilled water. The remaining crystal violet was eluted by adding 2 ml of ethanol and subsequently quantified by measuring absorbance at 570 nm. All assays were carried out in triplicate.

## Results

### Isolation of *P. migulae* strain HY-2 capable of growing on PHBA as the sole carbon source

Taxonomic analysis revealed that strain HY-2 was the most closely related to *P. migulae* strain 8R6 (GenBank accession number CP093428), with 99.8% 16S rRNA gene sequence identity and 90.92% ANI (Table S1, available in the online Supplementary Material), identifying it as a new strain within the *P. migulae* clade. Although strain HY-2 was isolated from a phenanthrene-grown enrichment culture, it did not exhibit apparent growth with phenanthrene as the sole carbon source. Instead, strain HY-2 demonstrated the ability to grow on PHBA as the sole carbon source, which is a potential biodegradation product of phenanthrene and which is often detected as an environmental pollutant derived from industrial activities [34,35]. Strain HY-2 was additionally confirmed to grow at 10 °C and exhibited weak growth even at 4 °C, while the optimal growth temperature was 30 °C, and no growth was observed at 40 °C – evaluated by observing bacterial colonies on LB agar plates.

### PHBA biodegradation ability of strain HY-2 at cold temperature

**Fig. 1.**
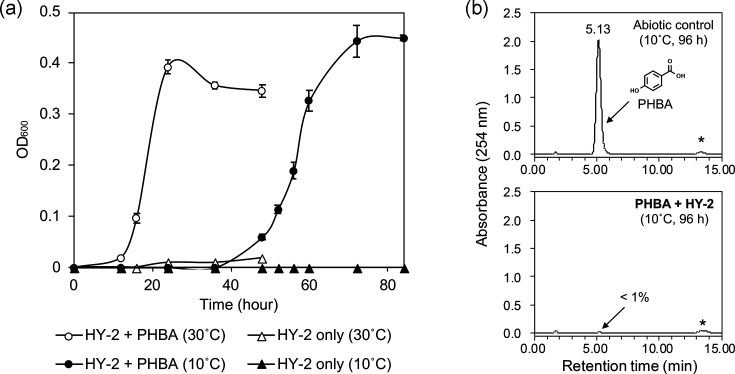
(**a**) Growth curve of *P. migulae* strain HY-2 incubated with 500 mg l^−1^ PHBA as the sole carbon source at either 30 °C (white circles) or 10 °C (black circles). Control cultures without a carbon source are also shown (white triangles, 30 °C; black triangles, 10 °C). Error bars represent sd (*n*=3). (**b**) HPLC analysis of cultures incubated with 500 mg l^−1^ PHBA at 10 °C for 96 h, with strain HY-2 cells (lower panel) or without cells (upper panel). *p-coumaric acid internal standard.

Strain HY-2 was confirmed to grow on 500 mg l^−1^ PHBA as the sole carbon source, exhibiting an exponential increase in cell density within 12 h and reaching stationary phase after around 24 h when cells were incubated at 30 °C. When cells were incubated at 10 °C, a longer lag phase (<48 h) was observed, and cells reached the stationary phase after around 72 h (Fig. 1a). After 96 h of incubation at 10 °C, >99% of the PHBA in the cultures (500 mg l^−1^) was biodegraded, as confirmed by HPLC analysis (Fig. 1b).

### Genomic characterizations of strain HY-2

**Fig. 2.**
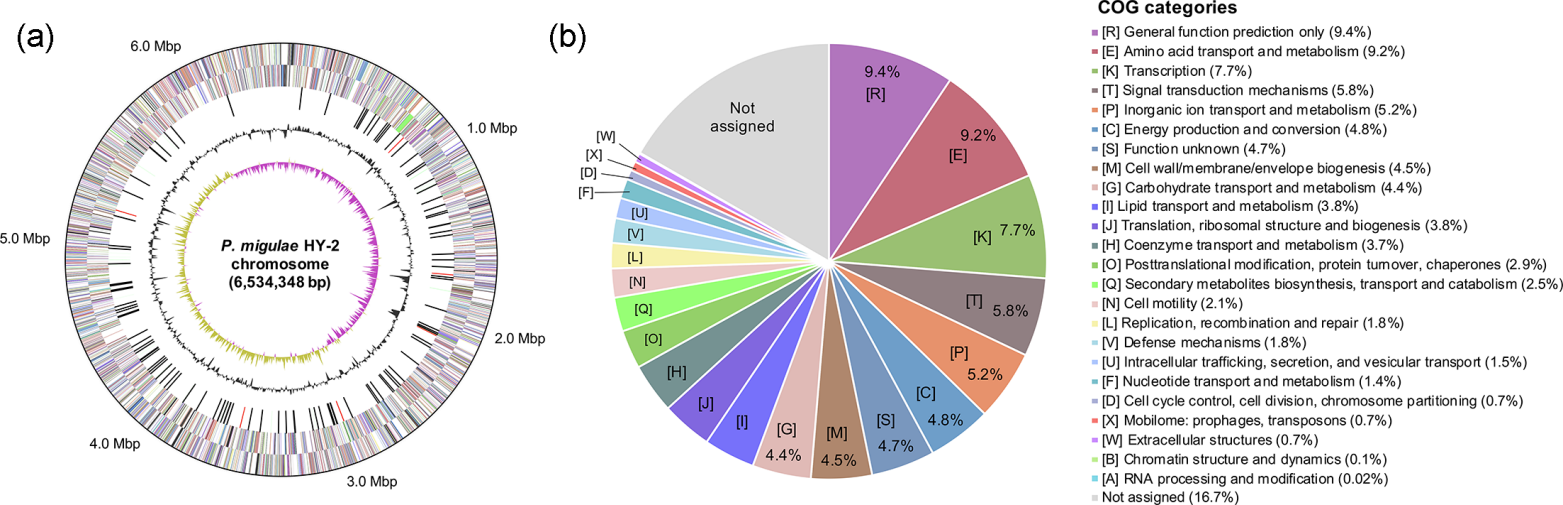
Complete genome sequence of *P. migulae* strain HY-2 with functional gene annotation. (**a**) Circular map of the strain HY-2 chromosome. Rings from outside to centre indicate predicted genes on the forward and reverse strand (coloured by COG classifications as listed in the panel (b), RNA genes (tRNAs, green; rRNAs, red; other RNAs, black), GC content (grey) and GC skew (yellow and purple). (**b**) Relative abundance of predicted functional genes in the strain HY-2 genome according to COG classification.

The complete genome of strain HY-2 was successfully obtained through *de novo* assembly of PacBio long-read sequencing data (Table S2), with 100% completeness as evaluated by CheckM. The strain HY-2 genome consisted of a single circular chromosome with a size of 6,534,348 bp, with its circularity confirmed by Bandage (Fig. S1), and contained no plasmids (Fig. 2). According to the IMG annotation pipeline, 5,922 coding sequences (CDSs), 19 rRNA genes and 70 tRNA genes were identified in the chromosome. Functional categorization of the predicted CDSs based on Clusters of Orthologous Groups (COG) classification revealed that the most abundant category was ‘[E] Amino acid transport and metabolism’ (9.2% of total CDSs), followed by ‘[K] Transcription’ (7.7%), ‘[T] Signal transduction mechanisms’ (5.8%) and ‘[P] Inorganic ion transport and metabolism’ (5.2%; Fig. 2).

### Predictions of functional genes involved in PHBA biodegradation in strain HY-2

**Fig. 3.**
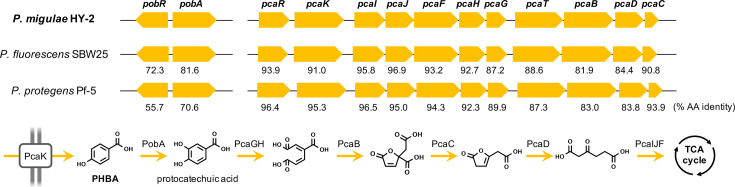
Predicted functional gene clusters for PHBA biotransformation in *P. migulae* strain HY-2 that are conserved in the genomes of other model *Pseudomonas* strains (*P. fluorescens* SBW25 and *P. protegens* Pf-5). Amino acid sequence identities (%) of each protein to those in strain HY-2 are presented.

Functional genes involved in aromatic hydrocarbon biodegradation in strain HY-2 were identified based on gene annotation pipeline results and comparative genomic analysis with other strains [36,37]. The genome of strain HY-2 was found to possess putative homologues of known functional genes responsible for PHBA biodegradation; these genes include *pobA* gene, encoding *p*-hydroxybenzoate 3-monooxygenase which converts PHBA to protocatechuic acid [38]; *pcaK* gene encoding *p*-hydroxybenzoate transporter; *pcaGH* genes encoding protocatechuate 3,4-dioxygenase responsible for the aromatic ring cleavage of protocatechuic acid [39,40]; and *pcaBCDFIJ* genes encoding enzymes for subsequent reactions in protocatechuic acid degradation, along with transcriptional regulator genes (*pobR* and *pcaR*) (Fig. 3). These functional genes were found as gene clusters similar to those previously characterized in other *Pseudomonas* strains [41], and the predicted proteins encoded in strain HY-2 exhibited 55–97% amino acid sequence identities to those in model organisms *P. fluorescens* SBW25 (GenBank accession AM181176) and *Pseudomonas protegens* Pf-5 (CP000076) (Fig. 3).

### Transcriptomic profiling of strain HY-2 degrading PHBA under cold stress

**Fig. 4.**
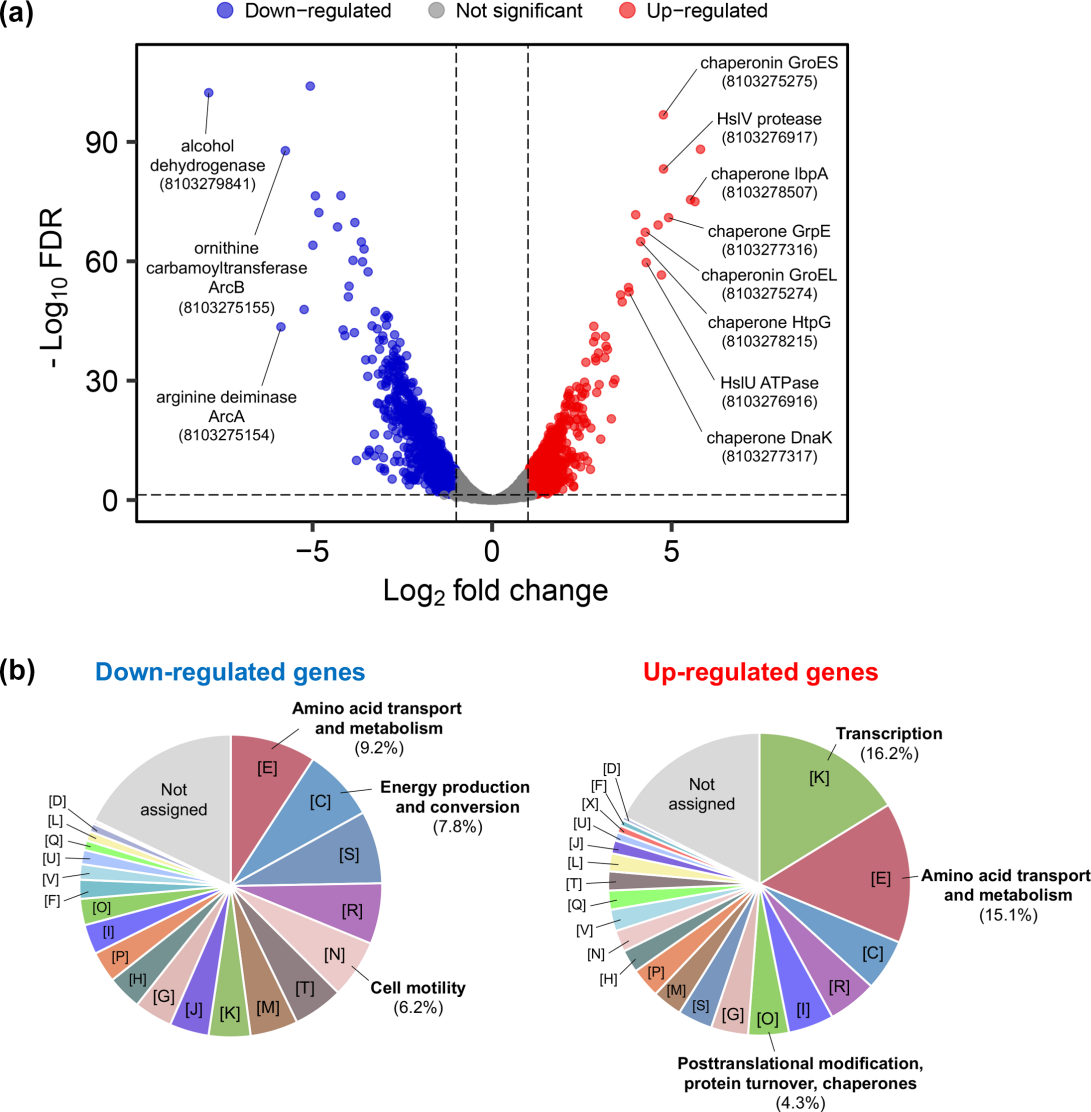
Transcriptomic profiling of *P. migulae* strain HY-2 in cultures grown at 30 °C or 10 °C on PHBA. (**a**) A volcano plot showing DEGs. Red dots indicate 783 genes that were significantly up-regulated at 10 °C (log_2_FC>1.0, FDR<0.05), while blue dots represent 860 genes that were significantly down-regulated at 10 °C (log_2_FC<−1.0, FDR<0.05) compared to 30 °C. Representative functional genes within the top ten most significantly up- or down-regulated genes are highlighted (IMG gene ID in parentheses). (**b**) Predicted functions of DEGs based on COG classification (coloured as shown in Fig. 2b). Relative abundances (%) of the most abundant or significantly enriched categories are presented: categories [E], [C] and [N] for down-regulated genes and categories [K], [E] and [O] for up-regulated genes.

The RNA-seq reads obtained from the strain HY-2 cultures were successfully mapped to the assembled genome, with an average mapping rate of 98.2%. Through the transcriptomic profiling, a total of 1,643 DEGs between cultures grown at 30 and 10 °C were identified, 783 genes were significantly up-regulated (log_2_FC>1.0, FDR<0.05) and 860 genes were significantly down-regulated (log_2_FC<−1.0, FDR<0.05) at 10 °C compared to 30 °C (Fig. 4a).

Among these up-regulated genes, genes categorized as the COG functional categories ‘[K] Transcription’ were the most abundant (127 genes, 16.2% of total up-regulated genes), which included various transcriptional regulator genes, followed by ‘[E] Amino acid transport and metabolism’ (118 genes, 15.1%). When compared to the abundances of these functional gene categories in the whole genome, the categories ‘[K]’ (2.11-fold enrichment among the up-regulated genes), ‘[E]’ (1.64-fold) and ‘[O] Posttranslational modification, protein turnover, chaperones’ (1.51-fold) appeared to be significantly more abundant among these up-regulated genes (Fig. 4b). The top ten most significantly up-regulated genes, excluding function-uncharacterized genes, included those encoding molecular chaperones IbpA (log_2_FC=5.527), GrpE (log_2_FC=4.915), HtpG (log_2_FC=4.137) and DnaK (log_2_FC=3.815), ATP-dependent protease-chaperone subunits (HslV, log_2_FC=4.773; HslU, log_2_FC=4.292) and chaperonin GroE small/large subunits (GroES, log_2_FC=4.769; GroEL, log_2_FC=4.265); these genes were all categorized as the category ‘[O]’ (Fig. 4a and Table 1). In the category ‘[K]’, three genes encoding CspA-family cold shock protein were identified to be up-regulated at 10 °C, with log_2_FC values of 1.216, 1.409 and 1.556, respectively.

**Table 1.** Most significantly up-regulated and down-regulated genes in strain HY-2 grown at 10 °C compared to 30 °C

IMG gene ID	Product	COG category	Log_2_ fold change	FDR
**Up-regulated genes**				
8103278507	IbpA, molecular chaperone	O	5.527	3.41×10^−76^
8103277316	GrpE, molecular chaperone	O	4.915	1.06×10^−71^
8103276917	HslV, ATP-dependent protease subunit	O	4.773	6.26×10^−84^
8103275275	GroES, chaperonin small subunit	O	4.769	1.59×10^−97^
8103277805	GSH-dependent disulphide-bond oxidoreductase	O	4.623	7.77×10^−70^
8103276916	HslU, ATP-dependent protease ATPase subunit	O	4.292	2.04×10^−60^
8103275274	GroEL, chaperonin large subunit	O	4.265	5.08×10^−68^
8103278215	HtpG, molecular chaperone	O	4.137	1.14×10^−65^
8103277317	DnaK, molecular chaperone	O	3.815	4.81×10^−53^
8103278011	methyl-accepting chemotaxis protein	T	3.622	1.61×10^−50^
**Down-regulated genes**				
8103279841	Alcohol dehydrogenase (cytochrome c)	G	−7.893	4.42×10^−103^
8103275155	ArcB, ornithine carbamoyltransferase	E	−5.759	1.68×10^−88^
8103275154	ArcA, arginine deiminase	E	−5.236	1.32×10^−48^
8103278410	Cytochrome c oxidase cbb3-type subunit III	C	−5.065	9.70×10^−105^
8103278411	Cytochrome c oxidase cbb3-type subunit IV	C	−4.992	9.53×10^−65^
8103278412	Cytochrome c oxidase cbb3-type subunit II	C	−4.922	3.74×10^−77^
8103274277	NAD-dependent aldehyde dehydrogenase	C	−4.826	5.91×10^−73^
8103274278	Alcohol dehydrogenase class IV	C	−4.305	2.34×10^−69^
8103279861	2-Oxoisovalerate dehydrogenase E2 component	C	−4.212	3.21×10^−77^
8103279860	2-Oxoisovalerate dehydrogenase E1 beta subunit	C	−4.100	4.78×10^−42^

Functional genes in the ‘[E]’ category were also determined as the most abundant among the down-regulated genes at 10 °C (72 genes, 9.2% of total down-regulated genes), followed by ‘[C] Energy production and conversion’ (61 genes, 7.8%). Besides, compared to the whole genome, the category ‘[N] cell motility’ showed the highest relative enrichment among the down-regulated genes (2.90-fold), which was followed by the ‘[C]’ category (1.63-fold, Fig. 4b). Consistent with this, the top ten most significantly down-regulated genes included those involved in energy production, such as subunits of cytochrome c oxidase (subunit II, log_2_FC=−4.922; subunit III, log_2_FC=−5.065; subunit IV, log_2_FC=−4.992), aldehyde dehydrogenase (log_2_FC=−4.826) and alcohol dehydrogenase (log_2_FC=−4.305, Table 1). Additionally, genes related to the assembly of the flagellar basal body/hook complex (*fli*, *flh* and *flg* genes) were found almost universally down-regulated at 10 °C (Table S3).

**Fig. 5.**
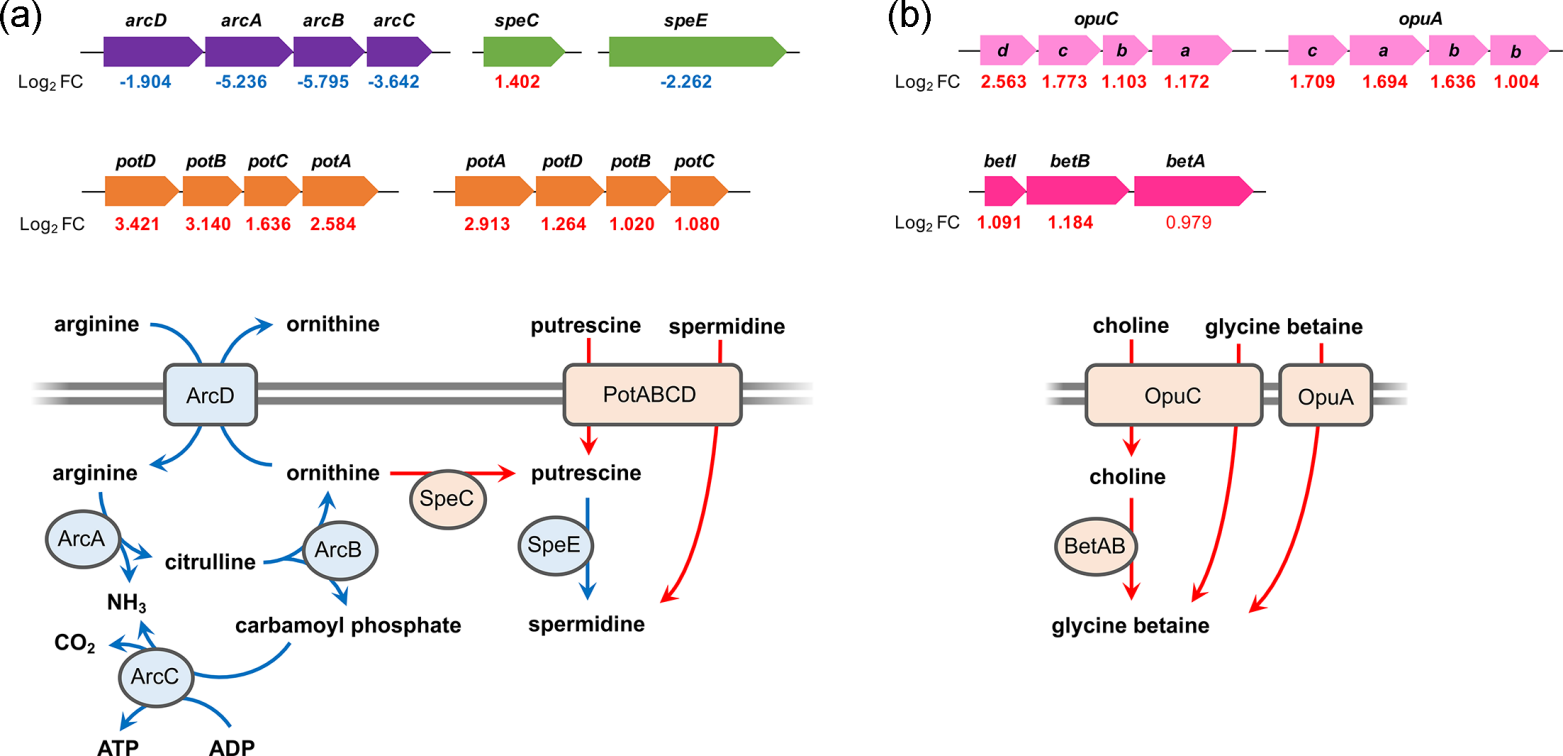
Selected differently expressed genes in *P. migulae* strain HY-2 at 10 °C involved in polyamine (spermidine/putrescine) accumulation (**a**) and glycine betaine accumulation (**b**). The upper panels show gene clusters with identified log_2_FC values, while the lower panels illustrate the predicted functions of the enzymes encoded by these gene clusters (red, up-regulated; blue, down-regulated).

Up-regulated genes categorized under ‘[E]’ included two sets of the *potABCD* gene operon (log_2_FC=1.020 to 3.421), which encodes the spermidine/putrescine transport system (PotA, ATP-binding protein; PotB and PotC, permease protein; PotD, substrate-binding protein) as well as the *speC* gene encoding ornithine decarboxylase, which converts ornithine to putrescine (log_2_FC=1.402; Table S4). The up-regulation of these genes suggests that cold temperature induced the uptake and intracellular accumulation of the polyamines spermidine and putrescine (Fig. 5a), although the *speE* gene, which encodes spermidine synthase that converts putrescine to spermidine, was down-regulated at 10 °C (log_2_FC=−2.262). Genes encoding ornithine carbamoyltransferase (*arcB*) and arginine deiminase (*arcA*) were among the most down-regulated genes (Table 1). Along with other contiguous genes, the genes of the *arcABCD* operon, which encodes enzymes responsible for the arginine deiminase system, were all down-regulated (log_2_FC=−1.904 to −5.759, Fig. 5a). Through the arginine deiminase system, ornithine produced from the conversion of arginine is exported in exchange for arginine via an arginine-ornithine antiporter encoded by *arcD*. Therefore, the down-regulation of the arginine deiminase system may further contribute to the accumulation of polyamines by retaining ornithine, the precursor of putrescine (Fig. 5a).

Additionally, the up-regulated genes under the ‘[E]’ category included the *opuC* gene operon, which encodes an osmoprotectant transport system responsible for the uptake of choline and glycine betaine (log_2_FC=1.00 to 1.71), as well as the *opuA* gene operon, which encodes a glycine betaine transport system (log_2_FC=1.06 to 1.61). Furthermore, the *betABI* operon, which encodes enzymes responsible for converting choline to glycine betaine, was also up-regulated, although the up-regulation of *betA* was not significant (*betA*, log_2_FC=0.979; *betB*, 1.184; *betI*, 1.091, Table S4). These findings suggest that intracellular accumulation of glycine betaine was induced under cold temperatures through both the uptake of glycine betaine and its precursor choline, as well as the conversion of choline to glycine betaine (Fig. 5b).

Regarding the functional genes predicted to be involved in PHBA biodegradation in strain HY-2 (*pob* and *pca* genes, Fig. 3), genes encoding the *p*-hydroxybenzoate transporter (*pcaK*, log_2_FC=1.937), the transcriptional regulator (*pcaR*, log_2_FC=1.430) and the glutaconate CoA-transferase subunit A (*pcaI*, log_2_FC=1.517) were up-regulated, while other genes were not identified as significantly differently expressed between two different growth temperatures (Table 2).

**Table 2.** Expression levels of PHBA-degrading genes in *P. migulae* strain HY-2 grown at 10 °C compared to 30 °C

IMG gene ID	Product	COG category	Log_2_ fold change	FDR
8103275250	PobR, transcriptional activator of *pobA*	K	0.287	1.75×10^−1^
8103275251	PobA, *p*-hydroxybenzoate 3-monooxygenase	C	−0.165	4.96×10^−1^
8103277853	PcaR, transcriptional regulator	K	1.430	3.98×10^−11^
8103277854	PcaK, *p*-hydroxybenzoate transporter	E	1.937	2.26×10^−20^
8103277855	PcaI, glutaconate CoA-transferase, subunit A	I	1.517	7.05×10^−12^
8103277856	PcaJ, glutaconate CoA-transferase, subunit B	I	0.997	6.82×10^−6^
8103277857	PcaF, 3-oxoadipyl-CoA thiolase	I	0.699	2.21×10^−3^
8103277858	PcaH, protocatechuate 3,4-dioxygenase, beta subunit	Q	0.577	8.41×10^−3^
8103277859	PcaG, protocatechuate 3,4-dioxygenase, alpha subunit	Q	0.711	1.26×10^−3^
8103277860	PcaT, dicarboxylic acid transporter	E	0.294	2.03×10^−1^
8103277861	PcaB, 3-carboxy-*cis,cis*-muconate cycloisomerase	F	0.189	4.13×10^−1^
8103277862	PcaD, 3-oxoadipate enol-lactonase	H	0.281	2.27×10^−1^
8103277863	PcaC, 4-carboxymuconolactone decarboxylase	R	0.310	1.64×10^−1^

### Effect of exogenous polyamines on biofilm formation

**Fig. 6.**
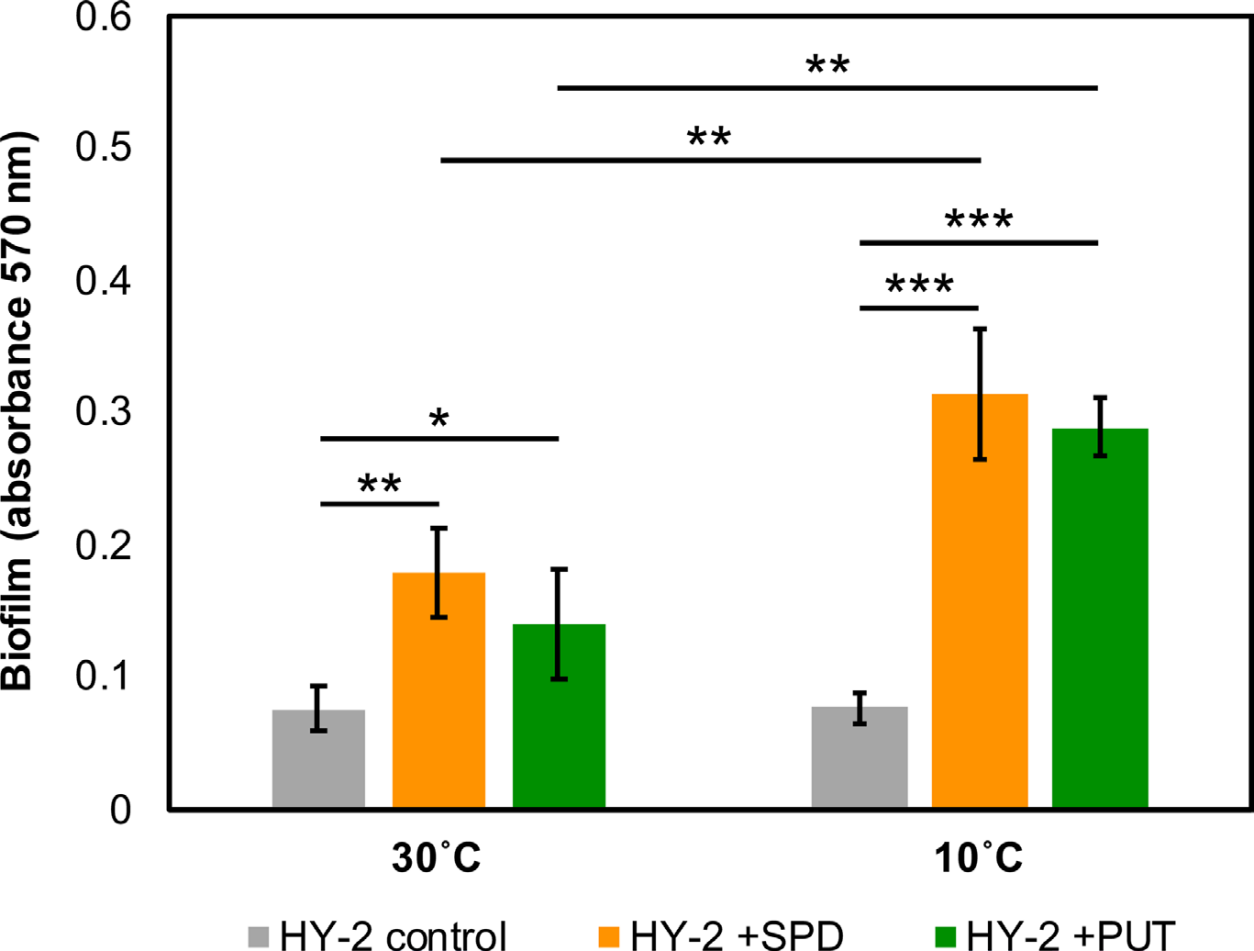
Enhanced biofilm formation of *P. migulae* strain HY-2 in response to exogenous polyamines. Biofilm formation was quantified in matured strain HY-2 cultures grown on PHBA at either 30 °C or 10 °C with 5 mM of spermidine (SPD, orange bars), putrescine (PUT, green bars) or without polyamines (grey bars). ***P<0.001, **P<0.01, *P<0.05 (Welch’s t-test); error bars represent sd (*n*=3).

A previous study reported that exogenous putrescine at a concentration of 5 mM promoted biofilm formation in *P. aeruginosa* via putrescine accumulation [42]. Therefore, the effects of exogenous polyamines, spermidine and putrescine, on the biofilm formation of strain HY-2 at either 30 °C or 10 °C were further examined by quantifying biofilm formation 24 h after reaching the stationary phase using the crystal violet biofilm assay. When strain HY-2 was cultured at 30 °C, exogenous spermidine and putrescine led to ~2.3-fold and 1.8-fold increases in biofilm formation, respectively (Fig. 6). While biofilm formation in strain HY-2 without exogenous polyamines did not differ significantly between 30 and 10 °C, exogenous polyamines had a more pronounced effect at 10 °C, resulting in ~4.1-fold (spermidine) and 3.8-fold (putrescine) increases compared to cultures grown at 10 °C without polyamines (Fig. 6).

## Discussion

**Fig. 7.**
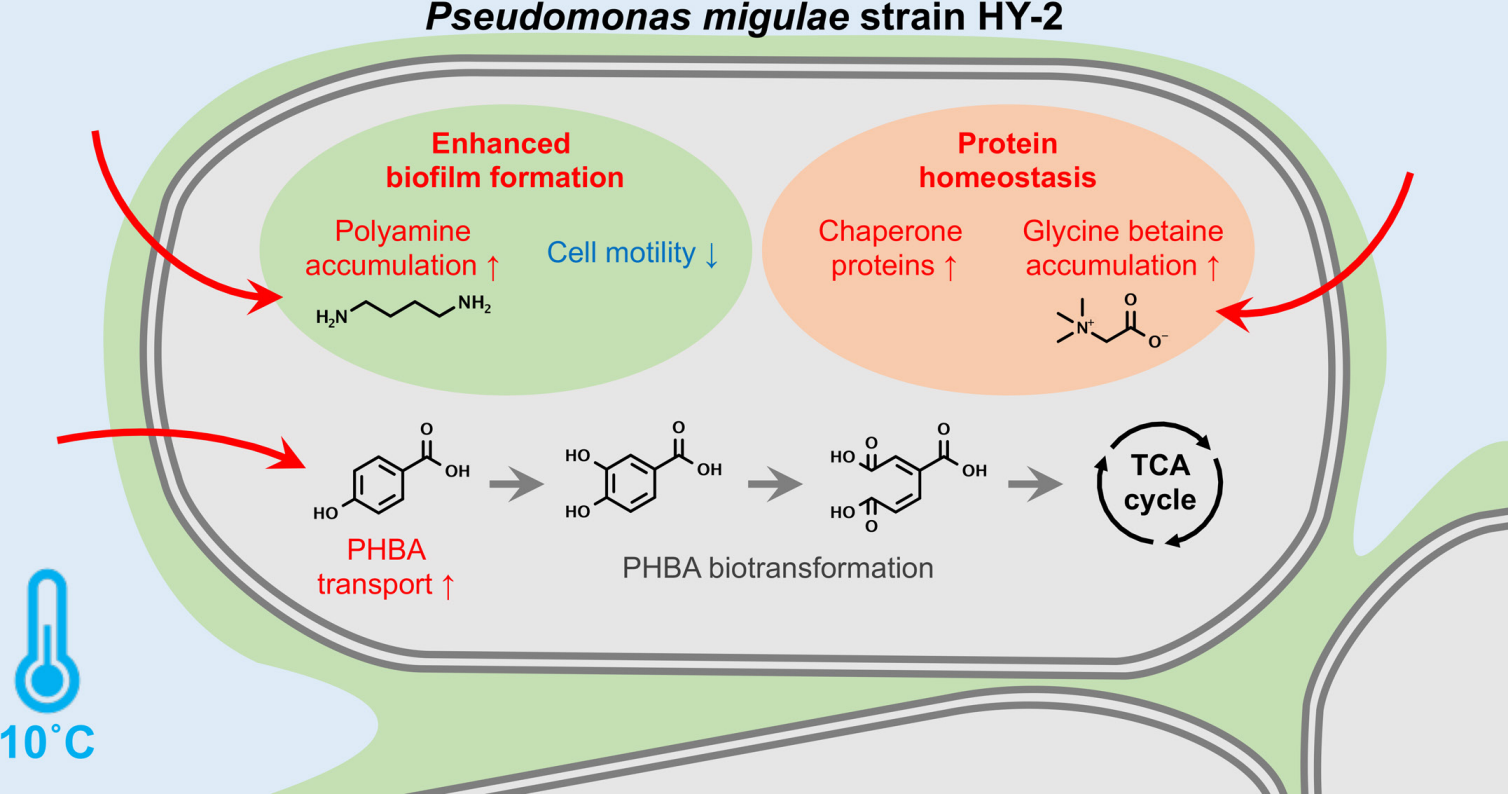
Schematic model summarizing the putative gene regulatory mechanisms in *P. migulae* strain HY-2 that support adaptation to cold stress while biodegrading the aromatic hydrocarbon pollutant PHBA. Under cold conditions, strain HY-2 employs multiple chaperones and the ‘chemical chaperone’ glycine betaine to help maintain protein homeostasis. Cold stress also up-regulates polyamine accumulation, which promotes stress-resistant biofilm formation. Despite these regulatory changes, the expression of genes involved in PHBA metabolism remains largely unaffected – except for the PHBA transporter – resulting in minimal impact on cell growth at non-optimal low temperatures.

*P. migulae* strain HY-2, which was shown to be capable of growing on PHBA under cold stress, was isolated from a bacterial consortium derived from groundwater outflow sediment that had been stored at 4 °C long term – this prolonged cold storage may have contributed to the enrichment of bacterial species with the ability to cope with cold stress. Transcriptomic profiling of train HY-2 revealed that multiple types of chaperones, IbpA, DnaK/DnaJ/GrpE, HslU/HslV, GroES/GroEL and HtpG, were among the most significantly up-regulated genes during growth on PHBA under cold stress (Fig. 4 and Table 1). These chaperones are universally conserved in *Pseudomonas* spp. (Table S5) and are well-characterized as bacterial heat shock proteins that respond to heat stress but have also been studied for their roles in resistance to various stress conditions, including cold and oxidative stresses. Chaperone IbpA (inclusion body-associated protein A) is known as Hsp20 (20 kDa heat shock protein) which prevents protein aggregation under oxidative stress [43,44]. Chaperone DnaK (Hsp70), along with its co-chaperones DnaJ and GrpE, protease-chaperone HslU/HslV, chaperonine GroES/GroEL (Hsp60/Hsp10 complex) and chaperone HtpG (Hsp90) have been previously characterized for their roles in resistance to cold stress in various bacterial genera [45,51]. In addition to the activities of these chaperones, which may contribute to post-translational modifications of proteins, CspA-family cold shock protein – an RNA chaperone known to be involved in transcriptional and translational regulation under cold stress [52] – was also up-regulated at 10 °C. Our results suggest that the simultaneous activity of various chaperones – at least ten types, which were identified as up-regulated and are housekeeping in *Pseudomonas* – may play a crucial role in maintaining protein homeostasis under cold stress (Fig. 7). Further bioassays using bacterial mutants or strains with suppressed expression of these chaperones shall help clarify the specific contributions of each chaperone to cold adaptation.

Cold stress also appeared to induce the uptake and accumulation of certain chemicals, polyamines (spermidine and putrescine) and glycine betaine in *P. migulae* strain HY-2 (Fig. 5). Glycine betaine has been characterized as a ‘chemical chaperone’ in bacteria, preventing protein aggregation under stress conditions in a chaperone-like manner [53,54]. In *Bacillus* and *Vibrio* strains, glycine betaine was identified as an effective cold stress protectant [55,56], suggesting it may also contribute to cold adaptation in *Pseudomonas*. Furthermore, spermidine and putrescine have also been reported as cytoprotective polyamines that enhance macromolecular functions by stabilizing the structure of DNA and RNA, as well as neutralizing toxic reactive oxygen species [57,58]. According to transcriptomic profiling, polyamine accumulation in strain HY-2 appeared to be induced through the simultaneous enhancement of polyamine uptake and synthesis, along with the suppression of consumption of its precursor ornithine via the arginine deiminase system (Fig. 5a), which has not previously been reported to the best of our knowledge. As previously reported, putrescine accumulation induces biofilm formation in *Pseudomonas*, likely due to an increased intracellular level of c-di-GMP, a known biofilm-promoting second messenger in *Pseudomonas* [42]. Our transcriptomic data also indicated that at least 26 genes involved in cell motility were significantly down-regulated at 10 °C (e.g. *fli*, *flh* and *flg* genes, Table S3), which may further contribute to the promotion of biofilm formation (Fig. 7); enhanced biofilm formation is considered to increase resistance to various environmental stresses in *Pseudomonas* [59]. Although exogenous polyamines were not provided to strain HY-2 during cultivation for transcriptomic analysis, subsequent bioassays using exogenous spermidine or putrescine confirmed enhanced biofilm formation in strain HY-2 at 10 °C compared to 30 °C (Fig. 6), supporting the transcriptomic analysis results. A gene encoding methyl-accepting chemotaxis protein was also identified as one of the most up-regulated genes at 10 °C (log_2_FC=3.622, Table 1), which was previously found to be up-regulated in *P. fluorescens* strain S01 during its growth at 15 °C, and was hypothesized to be related to its biofilm formation [16]. It remains uninvestigated whether polyamine-induced biofilm formation in strain HY-2 contributes to bacterial resistance to environmental stresses other than cold stress or how this function is conserved within the *Pseudomonas* genus – further comprehensive investigations using strain HY-2 and other *Pseudomonas* strains are needed to address these questions.

While cold stress in *P. migulae* strain HY-2 resulted in drastic changes in the regulation of certain genes involved in maintaining protein homeostasis and stress resistance, the expression of genes predicted to be responsible for PHBA biodegradation was mostly unaffected at 10 °C compared to 30 °C (Table 2). This suggests that the PHBA biodegradation function in strain HY-2 was retained under cold stress, at least at the transcriptional level, although the contribution of these functional genes to PHBA biodegradation still needs to be evidenced through further experiments, such as bioassays using gene knockout mutants. Interestingly, among genes within the PHBA-degrading gene cluster, the *pcaK* gene encoding a PHBA transporter was exceptionally significantly up-regulated at 10 °C (log_2_FC=1.937, Table 2), which may have facilitated acquisition of growth substrate (i.e. PHBA) while cell motility was suppressed under cold stress. When *Pseudomonas* bacteria encounter cold stress in natural environments, exogenous polyamines, spermidine and putrescine that are commonly supplied by surrounding other eukaryotic/prokaryotic organisms [60] are considered to enhance their biofilm formation, helping them to withstand the stress. This finding further suggests that supplementing exogenous polyamines may help stabilize the colonization of biotechnologically useful *Pseudomonas* strains, thereby maintaining or enhancing their effectiveness in pollutant bioremediation under even more severe or prolonged stress conditions.

## Conclusion

*P. migulae* strain HY-2 was revealed to be capable of utilizing the common aromatic hydrocarbon environmental pollutant PHBA while coping with cold stress through the employment of multiple chaperones and the ‘chemical chaperone’ glycine betaine, which are potentially involved in transcriptional, translational and post-translational regulation to maintain protein homeostasis under stress conditions (Fig. 7). Additionally, polyamine-induced biofilm formation appeared to be activated in response to cold stress, likely through coordinated up-regulation of polyamine uptake and biosynthesis, along with down-regulation of the arginine deiminase system and flagellar assembly. Besides the gene regulations supporting cold stress adaptation, the expression of genes responsible for PHBA biotransformation in strain HY-2 was found to be mostly unaffected and partly up-regulated under cold stress, resulting in minimal impact on the growth efficiency of strain HY-2 at non-optimal low temperatures. This study expands our understanding of gene regulatory properties in *Pseudomonas* species, offering insights into their resilience to environmental stresses and reevaluating their usability for bioremediation in natural environments without the need for excessive anthropogenic intervention.

## Supplementary material

10.1099/mgen.0.001470 Uncited Supplementary Material 1.

## Abbreviations

ANI: average nucleic acid identity
CDSs: coding sequences
COG: Clusters of Orthologous Groups
DEGs: differentially expressed genes
DMF: *N*,*N*-dimethylformamide
FDR: false discovery rate
LB: Luria-Bertani
log_2_FC: log_2_ fold change
PHBA: *p*-hydroxybenzoic acid
SBM: Stanier’s basal medium

## References

[1] Srivastava J, Naraian R, Kalra SJS, Chandra H (2014). Advances in microbial bioremediation and the factors influencing the process. Int J Environ Sci Technol.

[2] Kebede G, Tafese T, Abda EM, Kamaraj M, Assefa F (2021). Factors influencing the bacterial bioremediation of hydrocarbon contaminants in the soil: mechanisms and impacts. J Chem.

[3] Maberly SC, O’Donnell RA, Woolway RI, Cutler MEJ, Gong M (2020). Global lake thermal regions shift under climate change. Nat Commun.

[4] Phadtare S (2004). Recent developments in bacterial cold-shock response. Curr Issues Mol Biol.

[5] Chattopadhyay MK, Raghu G, Sharma YVRK, Biju AR, Rajasekharan MV (2011). Increase in oxidative stress at low temperature in an Antarctic bacterium. Curr Microbiol.

[6] Tribelli PM, López NI (2018). Reporting key features in cold-adapted bacteria. Life.

[7] Medić AB, Karadžić IM (2022). Pseudomonas in environmental bioremediation of hydrocarbons and phenolic compounds- key catabolic degradation enzymes and new analytical platforms for comprehensive investigation. World J Microbiol Biotechnol.

[8] Ivanova AA, Mullaeva SA, Sazonova OI, Petrikov KV, Vetrova AA (2022). Current research on simultaneous oxidation of aliphatic and aromatic hydrocarbons by bacteria of genus *Pseudomonas*. Folia Microbiol.

[9] Tomiyama Y, Takeshita T, Mori JF, Kanaly RA (2021). Functionalization of the model asphaltene 1-dodecylnaphthalene by *Pseudomonas aeruginosa* KK6 through subterminal metabolism. J Pet Sci Eng.

[10] Williams PA, Sayers JR (1994). The evolution of pathways for aromatic hydrocarbon oxidation in *Pseudomonas*. Biodegradation.

[11] Moreno R, Rojo F (2014). Features of pseudomonads growing at low temperatures: another facet of their versatility. Environ Microbiol Rep.

[12] de Sousa LP (2025). *Pseudomonas* from extreme environments: physiological and molecular adaptations. J Basic Microbiol.

[13] Weiser R, Green AE, Bull MJ, Cunningham-Oakes E, Jolley KA (2019). Not all *Pseudomonas aeruginosa* are equal: strains from industrial sources possess uniquely large multireplicon genomes. Microbial Genomics.

[14] Botelho J, Grosso F, Peixe L (2019). Antibiotic resistance in *Pseudomonas aeruginosa* - mechanisms, epidemiology and evolution. Drug Resist Updat.

[15] Frank S, Schmidt F, Klockgether J, Davenport CF, Gesell Salazar M (2011). Functional genomics of the initial phase of cold adaptation of *Pseudomonas putida* KT2440. FEMS Microbiol Lett.

[16] Song Q, Li X, Hou N, Pei C, Li D (2024). Chemotaxis-mediated degradation of PAHs and heterocyclic PAHs under low-temperature stress by *Pseudomonas fluorescens* S01: Insights into the mechanisms of biodegradation and cold adaptation. J Hazard Mater.

[17] Tsushima S, Kanaly RA, Mori JF (2023). Whole-genome sequence of *Periconia* sp. strain TS-2, an ascomycete fungus isolated from a freshwater outflow and capable of Mn(II) oxidation. *Microbiol Resour Announc*.

[18] Tsushima S, Nishi Y, Suzuki R, Tachibana M, Kanaly RA (2024). Formation of biogenic manganese oxide nodules on hyphae of a new fungal isolate of *Periconia* that immobilizes aqueous copper. Microbes Environ.

[19] Kunihiro M, Ozeki Y, Nogi Y, Hamamura N, Kanaly RA (2013). Benz[a]anthracene biotransformation and production of ring fission products by *Sphingobium* sp. strain KK22. Appl Environ Microbiol.

[20] Mori JF, Kanaly RA (2022). Natural chromosome-chromid fusion across rRNA operons in a *Burkholderiaceae* bacterium. Microbiol Spectr.

[21] Sakai M, Mori JF, Kanaly RA (2024). Assessment of bacterial biotransformation of alkylnaphthalene lubricating base oil component 1-butylnaphthalene by LC/ESI-MS(/MS). Chemosphere.

[22] Sakai M, Tomiyama Y, Mori JF, Kanaly RA (2022). Growth of *Sphingobium barthaii* KK22 on 1-ethylnaphthalene reveals diverse oxidative transformations and a complex metabolite profile. Int Biodeterior Biodegradation.

[23] Abe M, Kanaly RA, Mori JF (2023). Genomic analysis of a marine alphaproteobacterium *Sagittula* sp. strain MA-2 that carried eight plasmids. Mar Genomics.

[24] Kayama G, Kanaly RA, Mori JF (2022). Complete genome sequence of *Thalassospira* sp. strain GO-4, a marine bacterium isolated from a phenanthrene-enriched bacterial consortium. *Microbiol Resour Announc*.

[25] Kolmogorov M, Yuan J, Lin Y, Pevzner PA (2019). Assembly of long, error-prone reads using repeat graphs. Nat Biotechnol.

[26] Parks DH, Imelfort M, Skennerton CT, Hugenholtz P, Tyson GW (2015). CheckM: assessing the quality of microbial genomes recovered from isolates, single cells, and metagenomes. Genome Res.

[27] Wick RR, Schultz MB, Zobel J, Holt KE (2015). Bandage: interactive visualization of *de novo* genome assemblies. Bioinformatics.

[28] Jain C, Rodriguez-R LM, Phillippy AM, Konstantinidis KT, Aluru S (2018). High throughput ANI analysis of 90K prokaryotic genomes reveals clear species boundaries. Nat Commun.

[29] Chen S, Zhou Y, Chen Y, Gu J (2018). fastp: an ultra-fast all-in-one FASTQ preprocessor. Bioinformatics.

[30] Langmead B, Salzberg SL (2012). Fast gapped-read alignment with Bowtie 2. Nat Methods.

[31] Liao Y, Smyth GK, Shi W (2014). featureCounts: an efficient general purpose program for assigning sequence reads to genomic features. Bioinformatics.

[32] Robinson MD, McCarthy DJ, Smyth GK (2010). edgeR: a bioconductor package for differential expression analysis of digital gene expression data. Bioinformatics.

[33] Shao X, Xie Y, Zhang Y, Deng X (2019). Biofilm formation assay in *Pseudomonas syringae*. Bio-protoc.

[34] Lu P, Huang H, Sun Y, Qiang M, Zhu Y (2022). Biodegradation of 4-hydroxybenzoic acid by *Acinetobacter johnsonii* FZ-5 and *Klebsiella oxytoca* FZ-8 under anaerobic conditions. Biodegradation.

[35] Wang S, Bilal M, Hu H, Wang W, Zhang X (2018). 4-Hydroxybenzoic acid-a versatile platform intermediate for value-added compounds. Appl Microbiol Biotechnol.

[36] Kayama G, Kanaly RA, Mori JF (2022). Comprehensive genomic characterization of marine bacteria *Thalassospira* spp. provides insights into their ecological roles in aromatic hydrocarbon-exposed environments. Microbiol Spectr.

[37] Abe M, Sakai M, Kanaly RA, Mori JF (2025). Identification of a putative novel polycyclic aromatic hydrocarbon-biodegrading gene cluster in a marine *Roseobacteraceae* bacterium *Sagittula* sp. MA-2. *Microbiol Spectr*.

[38] Seibold B, Matthes M, Eppink MH, Lingens F, Van Berkel WJ (1996). 4-hydroxybenzoate hydroxylase from *Pseudomonas* sp. CBS3. Purification, characterization, gene cloning, sequence analysis and assignment of structural features determining the coenzyme specificity. Eur J Biochem.

[39] Fujisawa H, Hayaishi O (1968). Protocatechuate 3,4-dioxygenase: I. crystallization and characterization. J Biol Chem.

[40] Buchan A, Collier LS, Neidle EL, Moran MA (2000). Key aromatic-ring-cleaving enzyme, protocatechuate 3,4-dioxygenase, in the ecologically important marine *Roseobacter* lineage. Appl Environ Microbiol.

[41] Paliwal V, Raju SC, Modak A, Phale PS, Purohit HJ (2014). *Pseudomonas putida* CSV86: a candidate genome for genetic bioaugmentation. PLoS One.

[42] Liu Z, Hossain SS, Morales Moreira Z, Haney CH (2022). Putrescine and its metabolic precursor arginine promote biofilm and c-di-GMP synthesis in *Pseudomonas aeruginosa*. J Bacteriol.

[43] Matuszewska E, Kwiatkowska J, Kuczyńska-Wiśnik D, Laskowska E (2008). *Escherichia coli* heat-shock proteins IbpA/B are involved in resistance to oxidative stress induced by copper. Microbiology.

[44] Krajewski SS, Joswig M, Nagel M, Narberhaus F (2014). A tricistronic heat shock operon is important for stress tolerance of *Pseudomonas putida* and conserved in many environmental bacteria. Environ Microbiol.

[45] Susin MF, Baldini RL, Gueiros-Filho F, Gomes SL (2006). GroES/GroEL and DnaK/DnaJ have distinct roles in stress responses and during cell cycle progression in *Caulobacter crescentus*. J Bacteriol.

[46] Maillot NJ, Honoré FA, Byrne D, Méjean V, Genest O (2019). Cold adaptation in the environmental bacterium *Shewanella oneidensis* is controlled by a J-domain co-chaperone protein network. *Commun Biol*.

[47] Mayer MP (2021). The Hsp70-chaperone machines in bacteria. Front Mol Biosci.

[48] Di Pasqua R, Mauriello G, Mamone G, Ercolini D (2013). Expression of DnaK, HtpG, GroEL and Tf chaperones and the corresponding encoding genes during growth of *Salmonella* Thompson in presence of thymol alone or in combination with salt and cold stress. Food Res Int.

[49] Hossain MM, Nakamoto H (2002). HtpG plays a role in cold acclimation in cyanobacteria. Curr Microbiol.

[50] Tripathy S, Sen R, Padhi SK, Sahu DK, Nandi S (2014). Survey of the transcriptome of *Brevibacillus borstelensis* exposed to low temperature shock. Gene.

[51] Genest O, Wickner S, Doyle SM (2019). Hsp90 and Hsp70 chaperones: collaborators in protein remodeling. J Biol Chem.

[52] Rennella E, Sára T, Juen M, Wunderlich C, Imbert L (2017). RNA binding and chaperone activity of the *E. coli* cold-shock protein CspA. Nucleic Acids Res.

[53] Diamant S, Eliahu N, Rosenthal D, Goloubinoff P (2001). Chemical chaperones regulate molecular chaperones *in vitro* and in cells under combined salt and heat stresses. J Biol Chem.

[54] Bourot S, Sire O, Trautwetter A, Touzé T, Wu LF (2000). Glycine betaine-assisted protein folding in a lysA mutant of *Escherichia coli*. J Biol Chem.

[55] Ma Y, Wang Q, Gao X, Zhang Y (2017). Biosynthesis and uptake of glycine betaine as cold-stress response to low temperature in fish pathogen *Vibrio anguillarum*. J Microbiol.

[56] Hoffmann T, Bremer E (2011). Protection of *Bacillus subtilis* against cold stress via compatible-solute acquisition. J Bacteriol.

[57] Kumar S, Suyal DC, Yadav A, Shouche Y, Goel R (2020). Psychrophilic *Pseudomonas helmanticensis* proteome under simulated cold stress. Cell Stress Chaperones.

[58] Koh HY, Park H, Lee JH, Han SJ, Sohn YC (2017). Proteomic and transcriptomic investigations on cold-responsive properties of the psychrophilic Antarctic bacterium *Psychrobacter* sp. PAMC 21119 at subzero temperatures. Environ Microbiol.

[59] Craig K, Johnson BR, Grunden A (2021). Leveraging *Pseudomonas* stress response mechanisms for industrial applications. Front Microbiol.

[60] Chen D, Shao Q, Yin L, Younis A, Zheng B (2019). Polyamine function in plants: metabolism, regulation on development, and roles in abiotic stress responses. Front Plant Sci.

