# Supplementary material for: Transcriptomic profiling of Pseudomonas migulae revealed gene regulatory properties during biodegradation of aromatic hydrocarbons under cold stress
Source: Microb Genom. 2025 Sep 16;11(9):001470. doi: 10.1099/mgen.0.001470 (PMC12452175; doi:10.1099/mgen.0.001470)
Supplement: Uncited Supplementary Material 1. [file mgen-11-01470-s001.pdf]

Supplementary Information

Transcriptomic profiling of *Pseudomonas migulae* revealed gene regulatory properties during biodegradation of aromatic hydrocarbons under cold stress

Hiroki Yanagita, Robert A. Kanaly, and Jiro F. Mori

Graduate School of Nanobioscience, Yokohama City University, Japan

Table S1. Average nucleic acid identity (ANI) among *P. migulae* strain HY-2 and other *Pseudomonas* strains.

Table S2. Detailed information on PacBio long-read sequencing results.

Table S3. Expression levels of flagellar assembly genes in strain HY-2 grown at 10˚C compared to 30˚C.

Table S4. Expression levels of genes involved in polyamine and glycine betaine accumulation in strain HY-2 grown at 10˚C compared to 30˚C.

Table S5. Conservation of chaperone proteins in the genomes of strain HY-2 and representative *Pseudomonas* strains.

Figure S1. Visual validation of the circular chromosome of strain HY-2 using Bandage.

Table S1. Average nucleic acid identity (ANI) among *P. migulae* strain HY-2 and other *Pseudomonas* strains.

	**HY-2**	**8R6**	**CFML 95-321^T**	**SBW25**	**Pf-5**
***P. migulae* HY-2**		90.92%	90.90%	83.95%	83.96%
***P. migulae* 8R6**	90.92%		97.34%	83.81%	83.92%
***P. migulae* CFML 95-321^T**	90.90%	97.34%		83.73%	83.86%
***P. fluorescens* SBW25**	83.95%	83.81%	83.73%		83.46%
***P. protegens* Pf-5**	83.96%	83.92%	83.86%	83.46%	

Table S2. Detailed information on PacBio long-read sequencing results.

Number of reads	Average read length (bp)	Total read size (bp)	Assembly total size (bp)	Assembly GC content (%)	Assembly coverage
22,611	10,986	248,400,371	6,534,348	60.4	38.0

Table S3. Expression levels of flagellar assembly genes in strain HY-2 grown at 10˚C compared to 30˚C.

IMG gene ID	Product	COG category	Log_2 fold change	FDR
8103278127	FliE, flagellar hook-basal body complex protein	N	-2.328	1.88×10^{-27}
8103278128	FliF, flagellar M-ring protein	N	-2.670	1.29×10^{-30}
8103278129	FliG, flagellar motor switch protein	N	-2.697	1.00×10^{-23}
8103278130	FliH, flagellar assembly protein	N	-2.487	1.19×10^{-19}
8103278131	FliI, flagellum-specific ATP synthase	N	-2.047	8.47×10^{-17}
8103278132	FliJ, flagellar protein	N	-1.747	1.18×10^{-10}
8103278136	FliK, flagellar hook-length control protein	N	-1.064	8.27×10^{-7}
8103278137	FliL, flagellar protein	N	-2.340	3.75×10^{-23}
8103278138	FliM, flagellar motor switch protein	N	-2.740	6.39×10^{-38}
8103278139	FliN, flagellar motor switch protein	N	-2.291	2.91×10^{-18}
8103278140	FliO/FliZ, flagellar protein	N	-2.380	1.26×10^{-23}
8103278141	FliP, flagellar biosynthesis protein	N	-2.386	4.27×10^{-18}
8103278142	FliQ, flagellar biosynthesis protein	N	-1.534	6.78×10^{-6}
8103278143	FliR, flagellar biosynthesis protein	N	-1.730	1.22×10^{-9}
8103278144	FlhB, flagellar biosynthesis protein	N	-1.461	9.05×10^{-7}
8103278145	FlhA, flagellar biosynthesis protein	N	-2.592	1.24×10^{-25}
8103278146	FlhF, flagellar biosynthesis protein	N	-2.460	1.20×10^{-29}
8103278147	FlhG, flagellar biosynthesis protein	N	-2.229	9.16×10^{-24}
8103274998	FlgB, flagellar basal-body rod protein	N	-1.369	8.94×10^{-12}
8103274997	FlgC, flagellar basal-body rod protein	N	-1.685	2.12×10^{-15}
8103274996	FlgD, flagellar basal-body rod modification protein	N	-1.521	2.81×10^{-12}
8103274995	FlgE, flagellar hook protein	N	-0.907	4.44×10^{-5}
8103278095	FlgF, flagellar basal-body rod protein	N	-2.031	8.13×10^{-23}
8103278096	FlgG, flagellar basal-body rod protein	N	-2.445	6.54×10^{-28}
8103278097	FlgH, flagellar L-ring protein	N	-2.743	2.01×10^{-31}
8103278098	FlgI, flagellar P-ring protein	N	-2.651	3.63×10^{-25}
8103278099	FlgJ, peptidoglycan hydrolase	N	-2.163	9.13×10^{-17}

Table S4. Expression levels of genes involved in polyamine and glycine betaine accumulation in strain HY-2 grown at 10˚C compared to 30˚C.

IMG gene ID	Product	COG category	Log_2 fold change	FDR
8103275153	ArcD, arginine:ornithine antiporter / lysine permease	E	-1.904	4.15×10^{-7}
8103275154	ArcA, arginine deiminase	E	-5.236	1.32×10^{-48}
8103275155	ArcB, ornithine carbamoyltransferase	E	-5.759	1.68×10^{-88}
8103275156	ArcC, carbamate kinase	E	-3.642	1.38×10^{-65}
8103277367	SpeC, ornithine decarboxylase	E	1.402	1.29×10^{-10}
8103278590	PotD, spermidine/putrescine transport system substrate-binding protein	E	3.421	5.54×10^{-31}
8103278591	PotB, spermidine/putrescine transport system permease protein	E	3.140	1.70×10^{-36}
8103278592	PotC, spermidine/putrescine transport system permease protein	E	1.636	7.03×10^{-5}
8103278593	PotA, spermidine/putrescine transport system ATP-binding protein	E	2.584	6.45×10^{-20}
8103278604	PotC, spermidine/putrescine transport system permease protein	E	1.080	9.26×10^{-5}
8103278605	PotB, spermidine/putrescine transport system permease protein	E	1.020	7.34×10^{-6}
8103278606	PotD, spermidine/putrescine transport system substrate-binding protein	E	1.264	5.28×10^{-7}
8103278607	PotA, spermidine/putrescine transport system ATP-binding protein	E	2.913	9.38×10^{-28}
8103279697	SpeE, spermidine synthase	E	-2.262	3.86×10^{-29}
8103276089	BetI, transcriptional repressor of bet genes	K	1.091	1.87×10^{-8}
8103276090	BetB, betaine-aldehyde dehydrogenase	I	1.184	5.58×10^{-9}
8103276091	BetA, choline dehydrogenase	I	0.979	3.68×10^{-6}
8103277368	OpuCa, osmoprotectant transport system ATP-binding protein	E	1.172	4.53×10^{-7}
8103277369	OpuCb, osmoprotectant transport system permease protein	E	1.103	2.64×10^{-3}
8103277370	OpuCc, osmoprotectant transport system substrate-binding protein	E	1.773	1.68×10^{-6}
8103277371	OpuCd, osmoprotectant transport system permease protein	E	2.563	9.27×10^{-12}
8103279982	OpuAb, glycine betaine/proline transport system permease protein	E	1.004	1.64×10^{-3}
8103279983	OpuAb, glycine betaine/proline transport system permease protein	E	1.636	3.61×10^{-6}
8103279984	OpuAa, glycine betaine/proline transport system ATP-binding protein	E	1.694	3.80×10^{-13}
8103279985	OpuAc, glycine betaine/proline transport system substrate-binding protein	E	1.709	2.71×10^{-13}

Table S5. Conservation of chaperone proteins in the genomes of strain HY-2 and representative *Pseudomonas* strains.

**Bacterial strains (IMG Genome ID)**	**Chaperones (% amino acid identity to strain HY-2)**								
	IbpA	DnaK	DnaJ	GrpE	HslU	HslV	GroES	GroEL	HtpG
P. migulae HY-2 (8103273928)	+	+	+	+	+	+	+	+	+
P. protegens Pf-5 (637000220)	+ (88.5)	+ (96.1)	+ (96.0)	+ (93.6)	+ (92.8)	+ (96.6)	+ (93.8)	+ (94.1)	+ (95.9)
P. syringae Lz4W (2775507263)	+ (77.0)	+ (94.5)	+ (90.6)	+ (91.5)	+ (91.9)	+ (92.0)	+ (96.9)	+ (96.2)	+ (93.2)
P. fluorescens SBW25 (649633086)	+ (73.6)	+ (94.5)	+ (95.2)	+ (86.6)	+ (94.6)	+ (96.0)	+ (87.6)	+ (89.8)	+ (94.2)
P. putida KT2440 (637000222)	+ (81.8)	+ (90.6)	+ (88.8)	+ (69.2)	+ (89.9)	+ (92.6)	+ (91.8)	+ (88.8)	+ (98.1)
P. aeruginosa PAO1 (637000218)	+ (78.4)	+ (84.0)	+ (84.0)	+ (75.3)	+ (86.7)	+ (84.7)	+ (85.6)	+ (90.1)	+ (87.9)

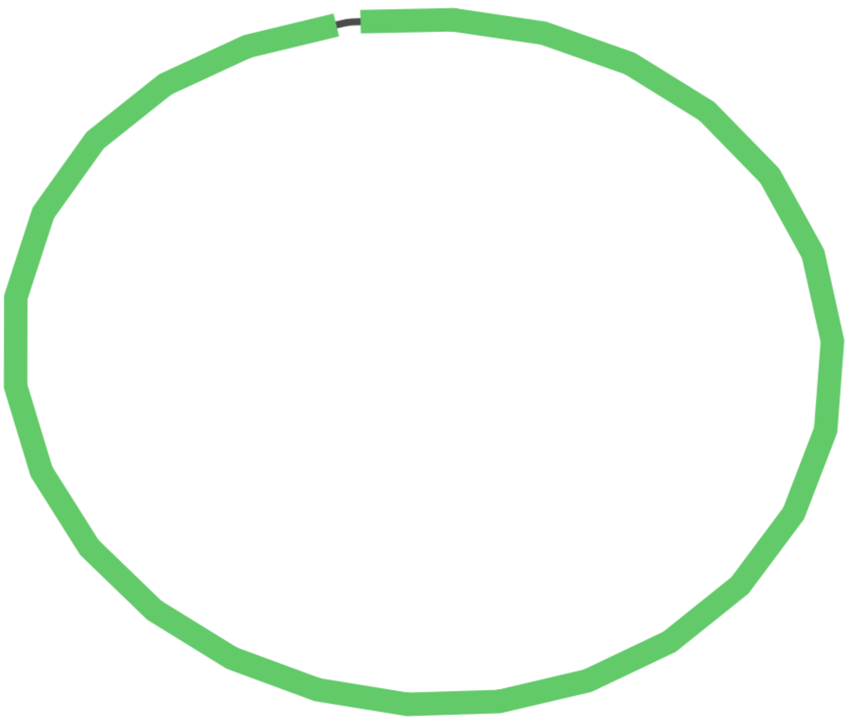

Figure S1. Visual validation of the circular chromosome of strain HY-2 using Bandage.
